# Expression of Cathepsins B, D, and G in Extracranial Arterio-Venous Malformation

**DOI:** 10.3389/fsurg.2021.676871

**Published:** 2021-08-02

**Authors:** Lauren Hansen, Helen D. Brasch, Erin Paterson, Josie Patel, Nicholas Bockett, Paul F. Davis, Swee T. Tan

**Affiliations:** ^1^Gillies McIndoe Research Institute, Wellington, New Zealand; ^2^Centre for the Study and Treatment of Vascular Birthmarks, Wellington Regional Plastic, Maxillofacial and Burns Unit, Hutt Hospital, Lower Hutt, New Zealand; ^3^Department of Surgery, The Royal Melbourne Hospital, The University of Melbourne, Melbourne, VIC, Australia

**Keywords:** arterio-venous malformation, cathepsin B, cathepsin D, cathepsin G, embryonic stem cells, mast cells

## Abstract

**Objectives:** We have previously identified a population of cells that expressed stemness-associated markers in extracranial arterio-venous malformation (AVM) and demonstrated expression of cathepsins B, D, and G on embryonic stem cell (ESC)-like populations in other vascular anomalies. This study investigated the expression of cathepsins B, D, and G, and their localization in relation to this primitive population in extracranial AVM.

**Methods:** Immunohistochemical staining was performed on AVM tissue samples from 13 patients to demonstrate expression of cathepsins B, D, and G. Western blotting was performed on four AVM tissue samples and three AVM-derived primary cell lines to confirm protein expression of cathepsins B and D proteins. RT-qPCR was performed on three AVM-derived primary cell lines to demonstrate transcript expression of cathepsins B, D, and G. Enzymatic activity assays were performed on three AVM-derived primary cell lines to investigate if cathepsins B and D were active. Localization of the cathepsins was investigated using immunofluorescence dual-staining of the cathepsins with the ESC markers OCT4 and SOX2, and mast cells marker chymase on two of the 13 AVM tissue samples.

**Results:** Immunohistochemical staining demonstrated expression of cathepsins B, D, and G in all 13 AVM tissue samples. Western blotting showed expression of cathepsins B and D proteins in all four AVM tissue samples and all three AVM-derived primary cell lines. RT-qPCR demonstrated transcripts of cathepsins B, D, and G in all three AVM-derived primary cell lines. Enzymatic activity assays showed that cathepsins B and D were active. Immunofluorescence staining showed expression of cathepsins B and D on the OCT4+/SOX2+ endothelium and media of the lesional vessels and cells within the stroma in AVM *nidus*. Cathepsin G was expressed on the chymase+ phenotypic mast cells.

**Conclusions:** This study demonstrated the novel finding of the expression of cathepsins B, D, and G in AVM. Cathepsins B and D were expressed by the primitive population, and cathepsin G was localized to mast cells, within the AVM *nidus*.

## Introduction

Following the distinction between (infantile) hemangioma and vascular malformations by Mulliken and Glowacki (1), research over the past 40 years has provided insights into the pathogenesis of some of these vascular anomalies. Arterio-venous malformation (AVM), which comprises 5–15% of all vascular malformations (2, 3), remains poorly understood (4). It consists of direct connections between high-flow arterial vessels and low-flow venous channels causing arterio-venous shunting (5). This tangle of aberrant arterial and venous channels—the central *nidus*—bypasses the normal capillary network (6). Blood flows through the veins with increased pressure, causing secondary dilatation, collateralization and vessel wall thickening (7).

Treatment of AVM is challenging with the standard treatment being complete surgical excision of the *nidus*, following pre-operative super-selective embolization in selected cases (8). More recently ethanol sclerotherapy and embolization with occlusive agents have been employed (9). It has been suggested that embolization and surgery may induce hypoxia and inflammation in the surrounding tissue, contributing to a pro-angiogenic environment and further stimulating AVM progression and recurrence (8, 10). Recurrent rates of 40–75% following surgical excision of AVM have been reported, and the risk of recurrence increases with the Schöbinger stage of the lesion (7, 11). We have reported a recurrence rate of 8.7% following surgical resection with or without pre-operative super-selective embolization (8).

The pathogenesis of AVM is poorly understood and has been proposed to result from errors of vasculogenesis during weeks 4–6 of gestation causing defects in vessel remodeling (7, 10, 12). Unlike vascular tumors, which are associated with increased endothelial cell turnover, AVM has been assumed to be “quiescent” (1). The dysregulation of pathways associated with angiogenesis and normal vascular development, namely the phosphatidylinositol 3-kinase (PI3K)/AKT and mechanistic target of rapamycin (mTOR) pathways, have been implicated in the pathogenesis of vascular malformations (13). The PI3K/AKT pathway, mediated by Ras or selected tyrosine kinase receptors, controls normal vascular development and angiogenesis via the generation of lipid products which are also involved in a number of other cellular processes, such as cell proliferation and survival and cytoskeletal re-organization (13–15). The mTOR complex lies downstream of PI3K signaling and regulates ribosomal biogenesis promoting cell growth, division, survival and motility (14–16). The PI3K/AKT/mTOR pathway is a major regulator of cell survival, and its dysregulation has been implicated in a range of cancers (13, 15, 16). Sirolimus, an mTOR inhibitor, targets this pathway and early research has demonstrated its effectiveness in the treatment for some vascular malformations (1, 14, 17, 18), implying that the endothelium in AVM is not “quiescent,” as previously assumed.

We have recently identified the presence of a population of cells in AVM that expressed stemness-associated markers (18) involved in induction of pluripotent stem cells (19). Given that ESCs in other disease states have been shown to be dysfunctional when mTOR is inactivated, as cellular proliferation and pluripotency systems are rendered defective, we speculate that mTOR inactivation may influence pluripotency of cells that express stemness-associated markers in AVM (20, 21). There is increasing evidence of the presence of primitive cells within other vascular anomalies (22), including infantile hemangioma (IH) (23), pyogenic granuloma (24), venous malformation (25), port-wine stain (26), verrucous venous malformation (27) and lymphatic malformation (28). The primitive population in IH (29), pyogenic granuloma (24) and venous malformation (30) have been shown to express components of the renin-angiotensin system (RAS).

There are many different types of proteases with over 800 protease genes present in the human genome (31), Mammalian proteases can be classified into five distinct classes: aspartic and metalloproteases, cysteine, serine and threonine proteases, based on their mechanisms of catalysis (32). Sub-classes of proteases can exist across the different broad classes; cathepsins, for example, include serine proteases, cysteine proteases or aspartyl proteases (33). Fifteen classes of cathepsins have been identified in humans to date, contributing to a vast range of physiological functions including digestion, blood coagulation, innate immunity, complement activation, apoptosis, vesicular trafficking, angiogenesis, proliferation, and metastasis, among others (33). While often beneficial and ubiquitous throughout the body, dysregulation of cathepsins can result in a range of pathologies as extensive as the aforementioned functions (33). Of relevance, the potent collagenase and elastase activity of some cathepsins may contribute to endothelial modification, implicating them in the pathogenesis of atherosclerosis, abdominal aortic aneurysm, and heart valve disease (34, 35).

Cathepsins B, D, and G constitute bypass loops of the RAS (36), and they have also been implicated in vascular pathologies. Cathepsin B contributes to neovascularization (37), as well as the neurodegenerative processes underscoring Alzheimer's disease, tauopathies and Parkinson's disease (38). An increase in cathepsin B expression is associated with atherosclerosis and aortic aneurysms (37), and elevated cathepsin D expression has been demonstrated in the endothelium of varicose veins (39). The proteolytic function of cysteine cathepsins such as cathepsin B precipitates extracellular matrix (ECM) degradation, thought to be the preeminent cause of these disorders (37).

Expression of the cysteine protease cathepsin B, the aspartyl protease cathepsin D and the serine protease cathepsin G are expressed by ESC-like cells in IH (40), and microvessels in Dupuytren's disease (41) and keloid disorder (42). In IH, cathepsin B is present in cells of both the endothelium of the microvessel and the interstitium, while cathepsins D and G are principally expressed by cells within the stroma (40). Cathepsins B and D are localized to OCT4+ endothelium as well as the smooth muscle layer of microvessels within Dupuytren's disease and cathepsin G is predominantly localized to OCT4+/chymase+ mast cells within the stroma (41). We have recently demonstrated expression of cathepsins B and D by the OCT4+ endothelium of the microvessels of keloid-associated lymphoid tissues, and cathepsins B, D, and G by the perivascular cells, within keloid lesions (42).

This study demonstrated the novel finding of the expression of cathepsins B, D, and G in AVM with cathepsins B and D expressed by the primitive population, and cathepsin G was localized to mast cells, within the AVM *nidus*.

## Materials and Methods

The aim of this study is to investigate the expression of cathepsins B, D and G in AVM, and their localization in relation to the primitive population we have recently identified on the endothelium and media of lesional vessels and cells within the stroma of AVM (18).

### Tissue Samples

Extracranial AVM tissue samples from seven female and six male patients, aged 17–65 (mean, 35.8) years (Supplementary Table 1) including those used in our previous study (18), were sourced from the Gillies McIndoe Research Institute Tissue Bank and used for this study, which was approved by the Central Health and Disability Ethics Committee (Ref. 13/CEN/130). Written informed consent was obtained from all patients.

### AVM-Derived Primary Cell Lines

Primary cell lines were derived from three available fresh AVM tissue samples from the original cohort of 13 patients. Fragments of tissue were initially encased in Matrigel (cat# 354234, Corning, Tewkesbury, MA, USA) in 24-well plates with an explant culture media comprised of Dulbecco' Modified Eagle Medium (DMEM) and Glutamax™ (cat# 10569010, Gibco, Rockford, IL, USA) supplemented with 2% penicillin-streptomycin (cat# 15140122, Gibco) and 0.2% gentamicin-amphotericin B (cat# R01510, Gibco). Once sufficient cell growth was achieved to support transfer to a monolayer culture, cells were extracted by dissolving the Matrigel with Dispase (cat#354235, Corning) and transferred to an adherent culture flask with media containing DMEM with Glutamax™ supplemented with 10% fetal bovine serum (cat#10091148, Gibco), 5% mTeSR™1 Complete Medium (cat#85850, STEMCELL Technologies, Vancouver, BC, Canada), 1% penicillin-streptomycin, and 0.2% gentamycin-amphotericin in a humidified incubator at 37°C and 5% CO_2_. Cells for the AVM-derived primary cell lines were expanded in culture and harvested between passages four and nine for use in Western blotting, RT-qPCR, and enzymatic activity assay analyses.

### Histochemical and Immunohistochemical Staining

Hematoxylin and eosin (H&E) staining was performed on 4μm-thick formalin-fixed paraffin-embedded sections of AVM samples from 13 patients. The presence of AVM was confirmed on H&E stained slides by an anatomical pathologist. Immunohistochemical staining of tissue sections with primary antibodies for cathepsin B (1:200; cat#NBP1-19797, Novus Biologicals, Centennial, CO, USA), cathepsin D (1:2000; cat#ab75852, Abcam, Cambridge, MA, USA), and cathepsin G (1:100; cat#NBP2-33498, Novus Biologicals), was performed on the Leica BOND^TM^ RX auto-stainer (Leica, Nussloch, Germany) using the BOND Polymer Refine Detection (cat#9800, Leica) with 3,3′-diaminobenzidine as the chromogen. Immunohistochemical-stained slides were mounted in Dako Mounting Medium (cat#CS703, Dako, Glostrup, Denmark). Positive human control tissues were placenta for cathepsin B, breast carcinoma for cathepsin D, and tonsil for cathepsin G. Negative control tissues were isotype controls for mouse (ready-to-use; cat#IR750, Dako, Copenhagen, Denmark) and rabbit (ready-to-use; cat#IR600, Dako).

### RT-qPCR

Total RNA was isolated from three available AVM-derived primary cell lines from the original cohort of 13 patients. From frozen cell pellets of 5 × 10^5^ viable cells, RNA was extracted using the RNeasy Micro kit protocol (cat#74004, Qiagen). An on-column DNase digest (cat#79254, Qiagen) step was included to remove potentially contaminating genomic DNA. RNA quantity was determined using a NanoDrop 2000 Spectrophotometer (Thermo Fisher Scientific, Waltham, MA, USA). Transcriptional expression was analyzed in triplicate using the Rotor-Gene Q (Qiagen), Rotor-Gene Multiplex RT-PCR Kit (cat#204974, Qiagen) and TaqMan Gene Expression Assay primer probes on 40 ng of RNA. The primer probes used were cathepsin B (Hs00157194_m1), cathepsin D (Hs00157205_m1) and cathepsin G (Hs01113415_g1) (cat#4331182, Thermo Fisher Scientific). Gene expression was normalized to the reference genes GAPDH (Hs99999905_m1) and PUM1 (Hs00206469_m1) (cat#4331182, Thermo Fisher Scientific). Universal human reference RNA (UHR; cat#CLT636690, Takara, Shiga, Japan), total RNA extracted from a range of healthy adult human tissues, was used as the calibrator for the 2^ΔΔ*Ct*^ analysis. Nuclease free water was added for the no template control and RNA from tonsil tissue was used as a positive control. A no reverse transcriptase control was included for the cathepsin G assay which has the potential to detect genomic DNA. The presence of the correctly sized bands from the endpoint amplification products were confirmed using 2% agarose gel electrophoresis (cat#G402002, Thermo Fisher Scientific) and imaged using the ChemiDoc MP (Bio-Rad, Hercules, CA, USA). Graphs were generated using GraphPad Prism (v8.0.2, San Diego, CA, USA) and results expressed as fold-change, relative to UHR. A fold-change cut off was set at 2.0 for up-regulated, and 0.5 for down-regulated, genes.

### Western Blotting

Total protein was extracted from four available snap-frozen AVM tissue samples and three AVM-derived primary cell lines from the original cohort of 13 patients. Tissue underwent pestle homogenization (cat#PES-15-B-SI, Corning) in ice-cold Radioimmunoprecipitation assay buffer (cat#89900, Pierce Biotechnology, Rockford, IL, USA) supplemented with a protease and phosphatase inhibitor cocktail (cat#78440, Pierce Biotechnology), cell lines were extracted as above without the homogenization step. Protein was quantified using a BCA assay (cat#23227, Pierce Biotechnology), and diluted in an equal volume of 2x LDS (cat#B0007, Invitrogen, Carlsbad, CA, USA). 20μg of total protein was separated by SDS-PAGE on 4-12% Bis-Tris gels (cat#NW04122BOX, Invitrogen) in MES SDS running buffer (cat#B0002, Invitrogen) and transferred to a PVDF membrane (cat#IB24001, Invitrogen) using an iBlot 2 (cat#IB21001, Thermo Fisher Scientific). Protein was detected on the iBind Flex (cat#SLF2000, Thermo Fisher Scientific), using primary antibodies for α-tubulin (1:2000; cat#62204; Thermo Fisher Scientific), cathepsin B (1:1000; cat#Ab58802, Abcam, Cambridge, United Kingdom) and cathepsin D (1:1000; cat#Ab75852; Abcam). Appropriate secondary antibodies used were anti-mouse Alexa Fluor 488 (1:1000; cat#A21202, Life Technologies) for α-tubulin, goat anti-mouse HRP (1:1000, cat#Ab6789, Abcam) for cathepsin B and goat anti-rabbit HRP (1:1000, cat#Ab6712, Abcam) for cathepsin D. α-Tubulin was used as a loading control to demonstrate even protein loading. The positive control for cathepsins B and D was snap-frozen human tonsillar tissue. A HepG2 cell line was used as an additional positive control for cathepsin D. To visualize HRP protein bands, Clarity Western ECL substrate (cat#1705061, Bio-Rad) was used with the ChemiDoc MP Imaging System (Bio-Rad) and Image Lab 6.0 software (Bio-Rad) to analyze protein bands.

### Immunofluorescence Staining

To confirm co-expression of two proteins, immunofluorescence staining was performed on two AVM tissue samples of the 13 patients included in immunohistochemical staining, using the same primary antibodies with the same concentrations and co-staining with either endothelial cell marker CD31 (ready-to-use, cat#PA0414, Leica), ESC markers OCT4 (1:30, cat#309M-16, Cell Marque) and SOX2 (1:500; cat#PA1-094, Thermo Fisher Scientific) as surrogate markers of the primitive population, or chymase (1:1500, cat#PA528317, Invitrogen). Cathepsin B primary antibody from rabbit (as detailed above) and mouse (cat#Ab58802, Abcam) Vectafluor Excel anti-mouse (ready-to-use; cat#VEDK2488, Vector Laboratories, Burlingame, CA, USA), and Vectafluor Excel anti-rabbit (ready-to-use; cat#VEDK1594, Vector Laboratories) were used for appropriate detection. All antibodies were diluted with BOND primary antibody diluents (cat#AR9352, Leica). Immunofluorescence-stained slides were mounted in Vectashield HardSet anti-fade mounting medium and counter-stained with 4′,6-diamidino-2-phenylindole. Appropriate secondary only negative controls were run for ESC markers OCT4 and SOX2, endothelial cell marker CD31, chymase, and cathepsin G. All immunofluorescence staining was performed on the Leica BOND^TM^ RX auto-stainer using a BOND Detection system (cat#DS9455, Leica).

### Image Capture and Analysis

Immunohistochemical-stained slides were viewed and the images were captured on the Olympus BX53 light microscope fitted with an Olympus SC100 camera (Olympus, Tokyo, Japan), and processed with cellSens 2.0 software (Olympus). Immunofluorescence-stained slides were viewed and imaged with an Olympus FV1200 biological confocal laser-scanning microscope and processed with cellSens Dimension 1.17 software (Olympus).

### Enzyme Activity Assays

Enzyme activity assays were performed on the three available AVM-derived primary cell lines used for western blot analysis using enzyme activity assay kits to determine enzymatic activity of cathepsin B (cat#ab65300, Abcam), and cathepsin D (cat#ab65302, Abcam). Assay procedure was carried out according to manufacturer protocol. Due to a very small dynamic range for the cathepsin D activity assay, a titration was carried out using a recombinant cathepsin D protein to validate the assay. A further titration assay was carried out using tissue and cell line samples to establish the appropriate amount of protein to add for a valid assay result. Fluorescence was measured in a black 96-well plate (cat#3631, Corning, ME, USA) using the Varioskan Flash plate reader (cat#MIB5250030, Thermo Fisher Scientific). Human tonsil tissue and HepG2 cell lysates were used as appropriate positive and denatured for use as negative controls.

## Results

### AVM Tissue Samples Demonstrated Characteristic Angioarchitecture of the *Nidus*

**Figure 1:**
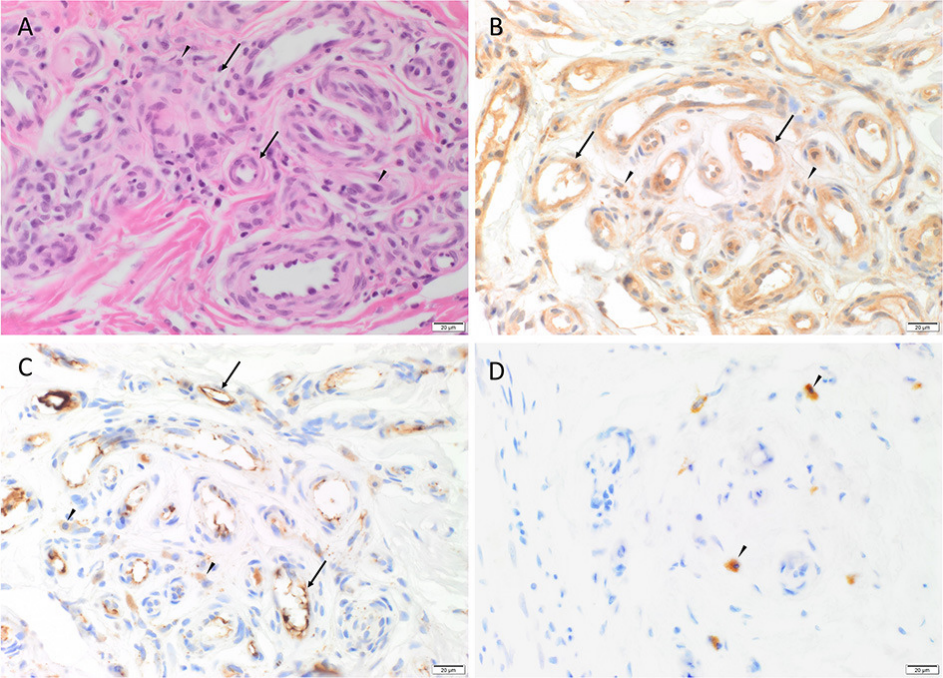
Representative H&E and immunohistochemical staining of arterio-venous malformation (AVM) tissue samples. H&E staining demonstrated AVM *nidus* consisting a tangle of vessels of varying luminal diameter and wall thickness (*arrows*), surrounded by a fibrous stroma (*arrowheads*) **(A)**. Immunohistochemical staining showed cytoplasmic expression of cathepsin B (**B**, brown), cathepsin D (**C**, brown) within the endothelium (*arrows*) of the lesional vessels and cells within the stroma (*arrowheads*), and cytoplasmic expression of cathepsin G (**D**, brown) in cells within the stroma (*arrowheads*). Nuclei were counterstained with hematoxylin (**B**–**D**, blue). Original magnification**:** 400x.

Hematoxylin and eosin (H&E) staining of all 13 AVM tissue samples demonstrated the *nidus* consisting a tangle of malformed thick-walled and dilated capillaries, arterioles and venules, within a background of fibrous stroma (Figure 1A).

### AVM Tissue Samples Expressed Cathepsins B, D, and G

Immunohistochemical staining demonstrated ubiquitous, granular and cytoplasmic staining of cathepsins B and D, in varying intensities in all 13 AVM tissue samples. Cathepsin B expression was generally stronger than that of cathepsin D. Cathepsin B was present on the endothelium and media of the lesional vessels more so than cells within the stroma in nine of the 13 cases, and equally in the remainder (Figure 1B). Staining of cathepsin D (Figure 1C) on the endothelium and media of lesional vessels was similar to the cells within the stroma. Cathepsin G was expressed by cells within the stroma with no staining on the lesional vessels (Figure 1D). The staining patterns of cathepsins B, D and G in all 13 cases of AVM are shown in Supplementary Table 2.

Positive staining was demonstrated on human tissues: placenta for cathepsin B (Supplementary Figure 1A), breast carcinoma for cathepsin D (Supplementary Figure 1B) and tonsil for cathepsin G (Supplementary Figure 1C). A negative stain using Flex Negative Control Mouse and Flex Negative Control Rabbit on a section of HPWS showed no staining, demonstrating that secondary and chromogenic detection were not signal producing (Supplementary Figure 1D).

### Cathepsins B and D Proteins Were Expressed by AVM Tissue Samples and AVM-derived Primary Cell Lines

**Figure 2:**
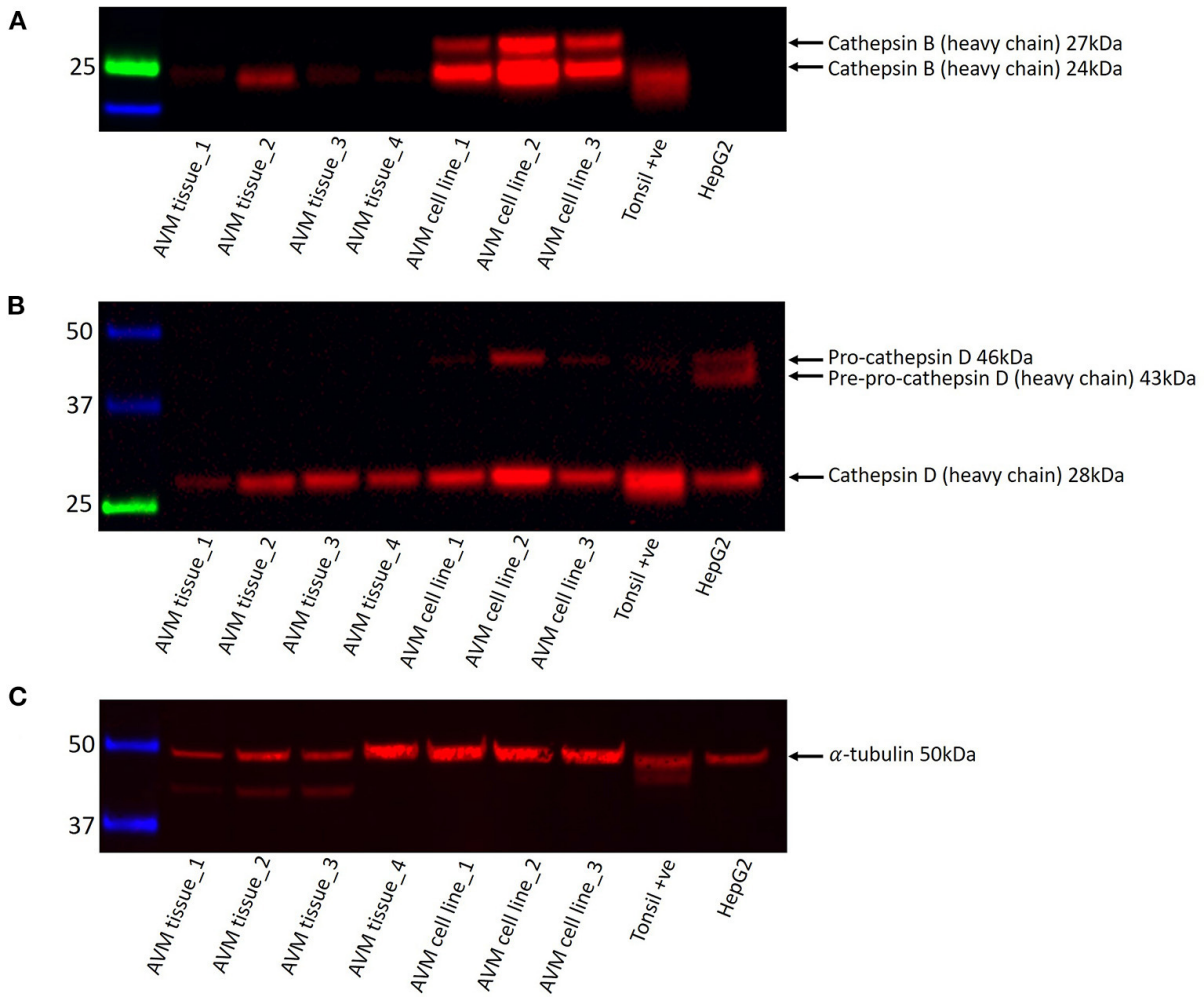
Representative western blot images of total protein extracted from four arterio-venous (AVM) tissue samples and three AVM-derived primary cell lines probed for cathepsins B and D. Cathepsin B **(A)**, cathepsin D **(B)**, and α-tubulin **(C)** were present at the appropriate molecular weights, as labeled.

Western blotting was performed on four AVM tissue samples and three AVM-derived primary cell lines to demonstrate protein expression of cathepsins B and D. Cathepsin B was expressed with a strong band representing the Cathepsin B heavy chain at 24kDa in one of the four AVM tissue samples and in all three AVM-derived primary cell lines and with a faint band in three of the four tissue samples; and an additional 27kDa heavy chain in all three cell lines (Figure 2A). Cathepsin D was expressed at 28kDa in three of the four tissue samples and all three cell lines (Figure 2B). Pro-cathepsin D and pre-pro-cathepsin D were present in the cell lines at 46kDa and 43kDa, respectively (Figure 2B). α-Tubulin (50kDa) confirmed approximate equal protein loading for both tissue samples and primary cell lines and tonsillar tissue was used as a positive control (Figure 2C).

### Cathepsins B, D, and G Transcripts Were Expressed by AVM-Derived Primary Cell Lines

**Figure 3:**
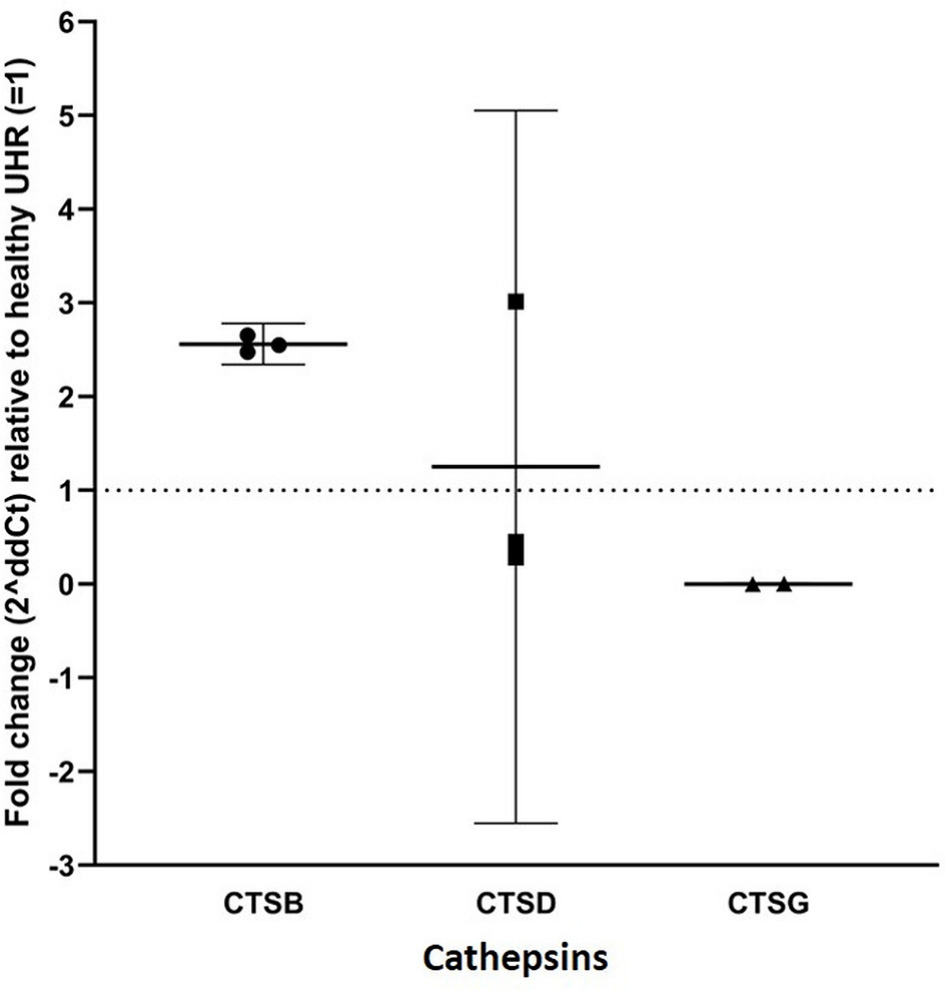
RT-qPCR on three arterio-venous malformation-derived primary cell lines. Cathepsin B (CTSB), cathepsin D (CTSD) and cathepsin G (CTSG) are shown on the X-axis. Expression was normalized to reference genes GAPDH and PUM1 and fold-change was calculated relative to healthy universal human reference (UHR), as indicated by the Y-axis. There was a 2.5-fold-change in expression of cathepsin B, an insignificant 1.25-fold change in cathepsin D expression, and significantly downregulated expression of cathepsin G, relative to UHR.

RT-qPCR performed on three AVM-derived primary cell lines confirmed mRNA expression of cathepsins B, D and G (Figure 3). Expression was normalized to reference genes GAPDH and PUM1 and fold-change calculated and expressed relative to the healthy UHR. Cathepsin B exhibited a 2.5-fold upregulation in expression, relative to UHR. Cathepsin D expression varied between the three cell lines, but there was on average, no biologically significant change, relative to the healthy UHR. Cathepsin G was expressed in two of the three cell lines and showed a more than 10-fold downregulation, relative to UHR.

Specific amplification of the PCR products was demonstrated using gel-electrophoresis, which confirmed detection of cathepsins B, D, and G amplicons at the expected molecular weights for the arterio-venous-derived primary cell lines (Supplementary Figures 2A–E), normalized to reference genes PUM1 (Supplementary Figure 2D) and GAPDH (Supplementary Figure 2E).

### Cathepsins B and D Were Present on the Endothelium of Lesional Vessels and Cells Within the Stroma, and Cathepsin G Was Expressed by Mast Cells

**Figure 4:**
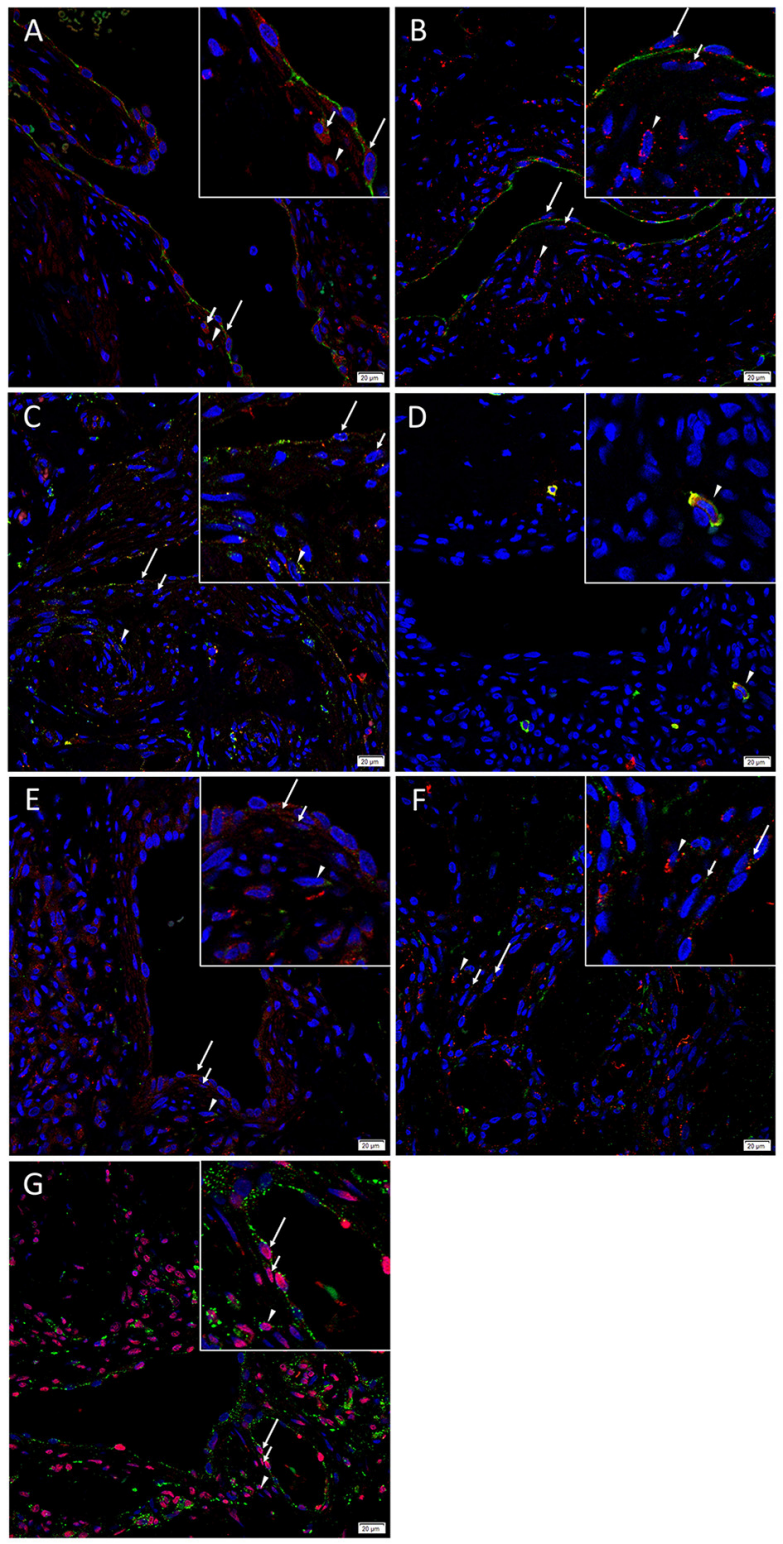
Representative immunofluorescence images of arterio-venous malformation tissue samples. Immunofluorescence staining showed expression of cathepsin B (**A**, red) and cathepsin D (**B**, red) on the CD31+ endothelium (**A,B**, green, *long arrows*) and media (*short arrows*) of the lesional vessels and cells within the stroma (*arrowheads*). Cathepsin B (**C**, green) and cathepsin D (**C**, red) were expressed on the endothelium (*long arrows*) and media (*short arrows*) of the lesional vessels and cells within and stroma (*arrowheads*). Cathepsin G (**D**, red) was expressed on the chymase+ (**D**, green) mast cells within the stroma (*arrowheads*). Cathepsin B (**E**, red), and cathepsin D (**F**, red) were expressed on the OCT4+ (**E**, **F**, green) endothelium (*long arrows*) and media (*short arrows*) of the lesional vessels and cells within the stroma (*arrowheads*). Cathepsin B (**G**, green) was expressed on the SOX2+ (**G**, red) endothelium (*long arrows*) and media (*short arrows*) of the lesional vessels and cells within the stroma (*arrowheads*). Cell nuclei were counterstained with 4′,6-diamidino-2-phenylindole (**A**–**G**, blue). Original magnification: 400x. The insets show enlarged views of the corresponding images.

Immunofluorescence staining was performed to demonstrate localization of cathepsins B, D, and G to different compartments of the AVM *nidus*. The CD31+ endothelium (Figures 4A,B, green) and media of the lesional vessels and cells within the stoma expressed cathepsin B (Figure 4A, red) and cathepsin D (Figure 4B, red). Cathepsin B exhibited more diffuse staining, and cathepsin D demonstrated focal staining. Both cathepsins were granular and localized to the cytoplasm. There was moderate co-expression of cathepsin B (Figure 4C, green) and cathepsin D (Figure 4C, red) on the endothelium and media of the lesional vessels and cells within the stroma. Cathepsin G (Figure 4D, red) was expressed by chymase+ (Figure 4D, green) cells within the stroma.

Immunofluorescence staining was carried out to determine whether the primitive population within AVM expressed cathepsins B and D, using OCT4 and SOX2 as surrogate markers. Dual-staining demonstrated that cathepsins B (Figure 4E, red) and cathepsin D (Figure 4F, red) were expressed on the OCT4+ (Figures 4E,F, green) cells on the endothelium and the media of the lesional vessels and cells within the stroma. Cathepsin B (Figure 4G, green) was expressed on the cytoplasm of cells that demonstrated nuclear expression of SOX2 (Figure 4G, red) in AVM tissue samples. Figure insets have been provided to show enlarged views of the corresponding images.

Supplementary Figures 3A–N present split images of stains in Figure 4. Negative control demonstrated minimal staining (Supplementary Figure 3O), confirming primary antibody specificity.

### Cathepsins B and D Within AVM-Derived Primary Cell Lines Were Enzymatically Active

**Figure 5:**
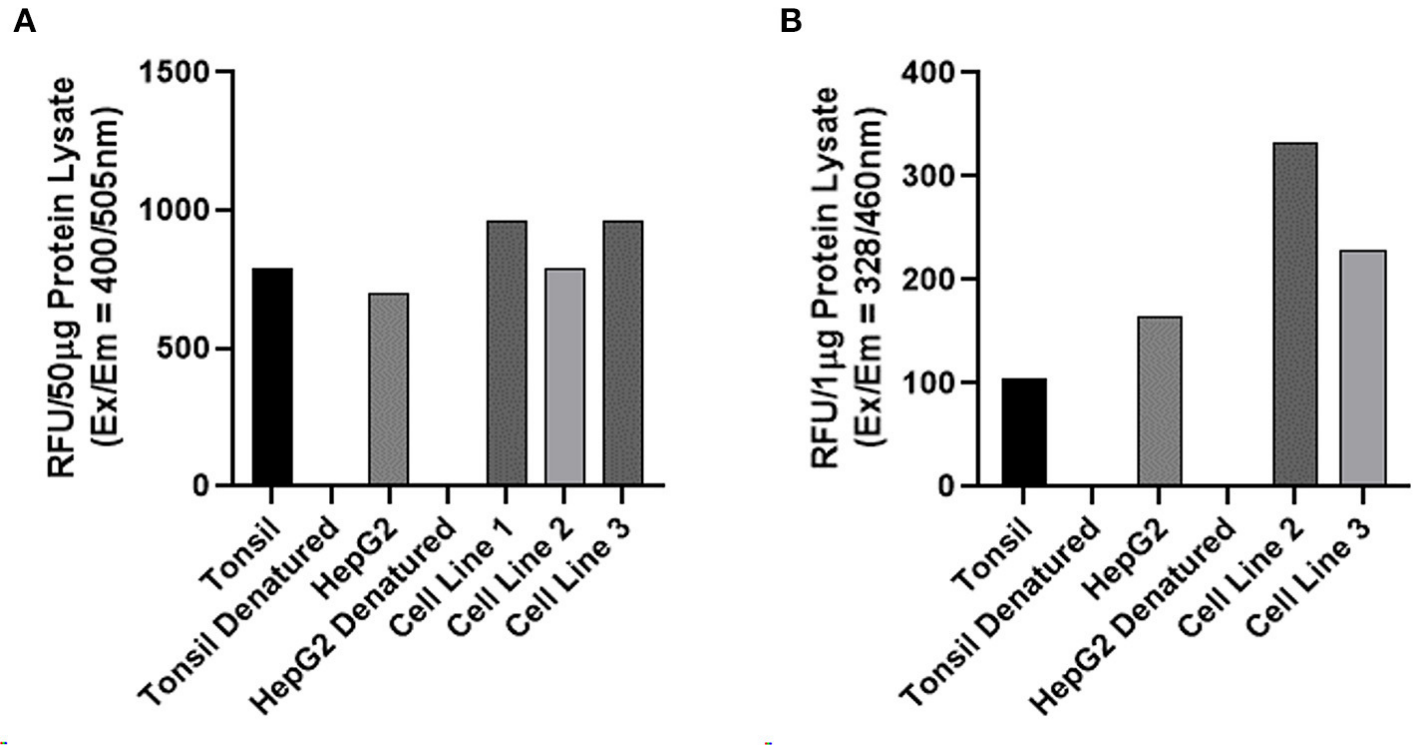
Enzymatic activity assays of on three arterio-venous malformation-derived primary cell lines for cathepsin B and cathepsin D. Enzymatic activity assays demonstrating enzymatic activity of cathepsin B **(A)** and cathepsin D **(B)** with tonsil tissue and HepG2 cells as positive controls and denatured tonsil and denatured HepG2 cells as negative controls.

Enzymatic activity assays performed on three AVM-derived primary cell lines demonstrated enzymatic activity for cathepsin B (Figure 5A) and cathepsin D (Figure 5B), with appropriate levels of activity detected for the positive and negative controls. Cathepsin D results were for 1 μg of protein. The titration validation work described in the Methods section is presented in Supplementary Figure 4.

## Discussion

AVM is a rare form of vascular malformation (43) that consists of direct arterio-venous communications (5) resulting in shunting of high-flow arterial blood into low-flow venous vessels (5) manifesting clinically as skin discoloration, pulsatility, bruit and thrill (5, 7, 44). Increased venous pressure causes vessel wall thickening, collateralization and venous dilation (45). Unlike IH (1), AVM grows commensurably with the child, with expansion during periods of hormonal stimulation, such as puberty and pregnancy (46). Current treatment of AVM is challenging and unsatisfactory.

The pathogenesis of AVM remains unclear, although it is believed to occur during weeks 4–6 of gestation as a result of errors in vasculogenesis causing disrupted vessel remodeling (7, 12). More recently, mutations affecting a number of pathways including the PI3K/AKT/mTOR pathway (13) have been identified in AVM, although treatments that target this pathway, such as sirolimus, produce inconsistent results (14, 17).

Cathepsins have been implicated in ECM degradation (37), endothelial cell migration and angiogenesis (47). The role of cathepsins has been investigated in cardiovascular and neurovascular diseases, and functional research has found inhibition of cathepsin to be an effective therapeutic target (47, 48). Murine models show that treatment with E-64d, a non-selective cysteine protease inhibitor, provides neuroprotection after trauma, functioning primarily through inhibition of cathepsin B (48, 49) and administration of NC-2300, a selective cystine protease inhibitor, reduces the incidence of cerebral aneurysm (50).

In this study, immunohistochemical and immunofluorescence staining demonstrated the presence of cathepsins B and D on the endothelium and media of the lesional vessels and cells within the stroma, and cathepsin G was expressed by cells within the stroma, of the *nidus* of AVM. These findings are confirmed at both the transcriptional and translational levels, by western blotting and RT-qPCR, respectively.

RT-qPCR demonstrated elevated expression of cathepsin B with a 2.5-fold-change and the expression of cathepsin D with a 0.3–3.0-fold-change, relative to UHR, across three AVM-derived primary cell lines. Cathepsin G expression was strongly downregulated, relative to UHR and was detected minimally in two cell lines and undetectable in the remaining cell line. This finding is consistent with the immunohistochemical and immunofluorescence results which showed diffuse expression of cathepsin B throughout the AVM tissues. The markedly lower expression of cathepsin G in the AVM-derived primary cell lines may be explained by the observation of its expression in scattered chymase+ mast cells within the AVM tissues.

Transcription factors OCT4, SOX2, NANOG, and c-MYC are involved in the induction and maintenance of induced pluripotent stem cells (19). We recently demonstrated the presence of a population of cells that express these stemness-associated markers within the *nidus* of AVM with nuclear and cytoplasmic expression of OCT4 and SOX2 on endothelium and media of the lesional vessels, and to a lesser extent, cells within the stroma (18).

In this study immunofluorescence dual-staining demonstrated ubiquitous expression of cathepsins B and D by the OCT4+, and cytoplasmic staining of cathepsin B within the SOX2+ cells (18). Because of species incompatibility we were unable to demonstrate if cathepsin D was also expressed by the SOX2+ cells also, although we were able to show extensive expression of cathepsin D by the cathepsin B+ cells that expressed SOX2. Immunofluorescence dual-staining demonstrated expression of cathepsin G by the chymase+ phenotypic mast cells within AVM tissues.

There is increasing evidence of the presence of ESC-like populations in vascular anomalies (22), including IH (23), pyogenic granuloma (24), venous malformation (25), verrucous venous malformation (27), lymphatic malformation (28), and port-wine stain (26). We have also demonstrated expression of components of the RAS by the stem cell population within a number of these vascular anomalies (24, 29, 30).

In the classical RAS, renin is released into the circulation either directly by juxtaglomerular cells in the kidney or as its precursor, pro-renin, extra-renally (51). Pro-renin binds to the pro-renin receptor (PRR) and undergoes a conformational change to become biologically active (51). While circulating renin is already active, it too can bind to PRR, of which enzymatic activity is increased 5-fold (51). Renin cleaves angiotensinogen to form angiotensin I (ATI), which is cleaved by angiotensin-converting enzyme (ACE) to form angiotensin II (ATII) (52). ATII is a biologically active peptide which acts directly on blood vessels by binding to angiotensin II receptor 1 (AT_1_R), causing vasoconstriction (52). Through its action on AT_1_R, ATII also activates growth pathways and cellular proliferation, and contributes to vascular hypertrophy, inflammatory responses and oxidative stress (53). Cathepsins B and D play a role in the activation of (pro)renin to form renin, which is involved in the cleavage of angiotensinogen to form ATI (36). Cathepsin G converts both angiotensinogen and ATI to form ATII, while chymase converts ATI to ATII (36).

The expression of the RAS by the ESC-like cells on the endothelium of IH underscores the observed accelerated involution of IH induced by β-blockers (54) and ACE inhibitors (55).

We have demonstrated expression of cathepsins B, D, and G by the ESC-like population in IH, with cathepsin B localized to the endothelium and cells within the stroma; cathepsin D being predominantly present on cells within the stroma, and cathepsin G expressed solely by cells within the stroma (40). Given that cathepsins B, D and G constitute bypass loops of the RAS, these findings may provide an explanation for the variable response to β-blockade and ACE inhibition in IH lesions (40).

Cathepsins are a sub-class of protease which, from their serine, cysteine and aspartyl groups, can be further classified into 15 different types, including B, D, and G (33, 35). Apart from their role in the RAS, some cysteine cathepsins play a key role in ECM degradation which contributes to the invasive properties of endothelial progenitor cells to promote neovascularization (56). The potent collagenase and elastase activity of some cathepsins, which affects endothelium, implicates them in the pathogenesis of a range of vascular pathology (35). Despite their increasing role in disease pathology, cathepsins are just some of over 800 proteases in the human body, and remain important in many normal physiological functions (31, 35).

There are existing inhibitors of cathepsin B (57, 58), cathepsin D (59, 60) and cathepsin G (61, 62), such as curcumin, an inhibitor of cathepsin B (63), pepstatin, an inhibitor of cathepsin D (64, 65) and chymostatin, an inhibitor of cathepsin G (66). Curcumin has been used in clinical trials for the treatment of chronic anterior uveitis (67), post-operative inflammation (68), and external cancerous lesions (69, 70). Further research is required to investigate the functional role of these cathepsins to determine their potential therapeutic effect on the primitive population in AVM.

## Data Availability Statement

The raw data supporting the conclusions of this article will be made available by the authors, without undue reservation.

## Ethics Statement

This study involving human participants was reviewed and approved by Central Health and Disability Ethics Committee (Ref. 13/CEN/130). Written informed consent to participate in this study was provided by the participants or their legal guardian/next of kin.

## Author Contributions

ST conceived the idea and designed the study. LH, HB, PD, and ST interpreted the immunohistochemical data. NB carried out confocal microscopy and performed the western blot analysis. LH, NB, and ST interpreted the immunofluorescence data. NB, LH, and ST interpreted the WB data. JP performed the RT-qPCR experiments and interpreted the data. EP performed enzymatic activity assays and cell culture experiments and interpreted the data. LH drafted the manuscript. ST critically revised the manuscript. All authors commented on and approved the manuscript.

## Conflict of Interest

ST and PD are inventors of a provisional patent Treatment of Vascular Anomalies PCT/NZ2017/050032, 2016; and Methods and Compositions for the Treatment of Hemangioma NZ761251, 2020. The remaining authors declare that the research was conducted in the absence of any commercial or financial relationships that could be construed as a potential conflict of interest.

## Publisher's Note

All claims expressed in this article are solely those of the authors and do not necessarily represent those of their affiliated organizations, or those of the publisher, the editors and the reviewers. Any product that may be evaluated in this article, or claim that may be made by its manufacturer, is not guaranteed or endorsed by the publisher.

## References

[1] Mulliken JB Glowacki J. Hemangiomas and vascular malformations in infants and children: a classification based on endothelial characteristics. Plast Reconstr Surg. (1982) 69:412–22. 10.1097/00006534-198203000-00002 7063565

[2] Sadick M Wohlgemuth WA Huelse R Lange B Henzler T Schoenberg SO. Interdisciplinary management of head and neck vascular anomalies: clinical presentation, diagnostic findings and minimalinvasive therapies. Eur J Radiol Open. (2017) 4:63–8. 10.1016/j.ejro.2017.05.001 28540347 PMC5432672

[3] Lee BB Baumgartner I Berlien HP Bianchini G Burrows P Do YS. Consensus document of the international union of angiology (IUA)-2013. Current concept on the management of arterio-venous management. Int Angiol. (2013) 32:9–36. 23435389

[4] Burrows PE. Biological approaches to the aggressive CVM lesion (antiangiogenic therapy). In: Kim Y-W, Lee B-B, Yakes WF, Do Y-S, editors. Congenital Vascular Malformations: A Comprehensive Review of Current Management. Berlin: Springer (2017). p. 343–7. 10.1007/978-3-662-46709-1_46

[5] Do YS Kim Y-W Lee B-B Yakes WF. Arteriovenous malformations (AVMs): clinical features and evaluation. In: Kim Y-W, Lee B-B, Yakes WF, Do Y-S, editors. Congenital Vascular Malformations: A Comprehensive Review of Current Management. Berlin: Springer (2017). p. 105–11. 10.1007/978-3-662-46709-1_17

[6] Bokhari MR SRA. B. Arteriovenous Malformation (AVM) of the Brain. Treasure Island, FL: StatPearls Publishing (2019). 28613495

[7] Kohout MP Hansen M Pribaz JJ Mulliken JB. Arteriovenous malformations of the head and neck: natural history and management. Plast Reconstr Surg. (1998) 102:643–54. 10.1097/00006534-199809030-00006 9727427

[8] Visser A FitzJohn T Tan ST. Surgical management of arteriovenous malformation. J Plast Reconstr Aesthet Surg. (2011) 64:283–91. 10.1016/j.bjps.2010.05.033 20663728

[9] Do YS Park KB. Endovascular treatment of AVM: trunk and extremity. In: Kim Y-W, Lee B-B, Yakes WF, Do Y-S, editors. Congenital Vascular Malformations: A Comprehensive Review of Current Management. Berlin: Springer (2017). p. 233–9. 10.1007/978-3-662-46709-1_33

[10] Fowell C Jones R Nishikawa H Monaghan A. Arteriovenous malformations of the head and neck: current concepts in management. Br J Oral Maxillofac Surg. (2016) 54:482–7. 10.1016/j.bjoms.2016.01.034 27020371

[11] Hartzell LD Stack BC Jr Yuen J Vural E Suen JY. Free tissue reconstruction following excision of head and neck arteriovenous malformations. Arch Facial Plast Surg. (2009) 11:171–7. 10.1001/archfaci.2009.6 19451451

[12] Suami H Lee B-B. Embryological background of congenital vascular malformations. In: Kim Y-W, Lee B-B, Yakes WF, Do Y-S, editors. Congenital Vascular Malformations: A Comprehensive Review of Current Management. Berlin: Springer (2017). p. 7–14. 10.1007/978-3-662-46709-1_2

[13] Couto JA Huang AY Konczyk DJ Goss JA Fishman SJ Mulliken JB. Somatic MAP2K1 mutations are associated with extracranial arteriovenous malformation. Am J Hum Genet. (2017) 100:546–54. 10.1016/j.ajhg.2017.01.018 28190454 PMC5339083

[14] Adams DM Trenor CC Hammill AM Vinks AA Patel MN Chaudry G. Efficacy and safety of sirolimus in the treatment of complicated vascular anomalies. Pediatrics. (2016) 137:e20153257. 10.1542/peds.2015-3257 26783326 PMC4732362

[15] Mendoza MC Er EE Blenis J. The Ras-ERK and PI3K-mTOR pathways: cross-talk and compensation. Trends Biochem Sci. (2011) 36:320–8. 10.1016/j.tibs.2011.03.006 21531565 PMC3112285

[16] Vignot S Faivre S Aguirre D Raymond E. mTOR-targeted therapy of cancer with rapamycin derivatives. Ann Oncol. (2005) 16:525–37. 10.1093/annonc/mdi113 15728109

[17] Chelliah MP Do HM Zinn Z Patel V Jeng M Khosla RK. Management of complex arteriovenous malformations using a novel combination therapeutic algorithm. JAMA Dermatol. (2018) 154:1316–9. 10.1001/jamadermatol.2018.3039 30326494 PMC6248124

[18] Luke Krishnan CS Brasch HD Patel J Bockett N Paterson E Davis PF. Stemness-associated markers in extracranial arterio-venous malformation. Front Surg. (2021) 8:621089 10.3389/fsurg.2021.621089 33816543 PMC8017302

[19] Takahashi K Yamanaka S. Induction of pluripotent stem cells from mouse embryonic and adult fibroblast cultures by defined factors. Cell. (2006) 126:663–76. 10.1016/j.cell.2006.07.024 16904174

[20] Aarts M Georgilis A Beniazza M Beolchi P Banito A Carroll T. Coupling shRNA screens with single-cell RNA-seq identifies a dual role for mTOR in reprogramming-induced senescence. Genes Dev. (2017) 31:2085–98. 10.1101/gad.297796.117 29138277 PMC5733499

[21] Zhou J Li D Wang F. Assessing the function of mTOR in human embryonic stem cells. In: Weichhart T, editor. mTOR: Methods and Protocols. Totowa, NJ: Humana Press (2012). p. 361–72. 10.1007/978-1-61779-430-8_23 22125078

[22] Kilmister EJ Hansen L Davis PF Hall SRR Tan ST. Stemness-associated markers are expressed in vascular anomalies. Front Surg. (2021) 7:610758. 10.3389/fsurg.2020.610758 33634164 PMC7900499

[23] Itinteang T Tan ST Brasch HD Steel R Best HA Vishvanath A. Infantile haemangioma expresses embryonic stem cell markers. J Clin Pathol. (2012) 65:394–8. 10.1136/jclinpath-2011-200462 22447921

[24] Papali'i-Curtin JC Brasch HD van Schaijik B de Jongh J Marsh RW Tan ST. Expression of components of the renin-angiotensin system in pyogenic granuloma. Front Surg. (2019) 6:13. 10.3389/fsurg.2019.00013 31024924 PMC6465765

[25] Tan EMS Siljee SD Brasch HD Enriquez S Tan ST Itinteang T. Embryonic stem cell-like subpopulations in venous malformation. Front Med. (2017) 4:162. 10.3389/fmed.2017.00162 29046873 PMC5632722

[26] Williams J Brasch HD Bockett N Patel J Paterson E Davis PF. Embryonic stem cell-like population in hypertrophic port-wine Stain. J Vasc Anom. (2021) 2:e006. 10.1097/JOVA.0000000000000006

[27] Laing EL Brasch HD Steel R Jia J Itinteang T Tan ST. Verrucous hemangioma expresses primitive markers. J Cutan Pathol. (2013) 40:391–6. 10.1111/cup.12078 23379586

[28] Eady EK Brasch HD de Jongh J Marsh RW Tan ST Itinteang T. Expression of embryonic stem cell markers in microcystic lymphatic malformation. Lymphat Res Biol. (2019) 17:496–503. 10.1089/lrb.2018.0046 30901291

[29] Itinteang T Brasch HD Tan ST Day DJ. Expression of components of the renin-angiotensin system in proliferating infantile haemangioma may account for the propranolol-induced accelerated involution. J Plast, Reconstr Aesth Surg. (2011) 64:759–65. 10.1016/j.bjps.2010.08.039 20870476

[30] Siljee S Keane E Marsh R Brasch HD Tan ST Itinteang T. Expression of the components of the renin-angiotensin system in venous malformation. Front Surg. (2016) 3:24. 10.3389/fsurg.2016.00024 27200356 PMC4853390

[31] Bond JS. Proteases: History, discovery, and roles in health and disease. J Biol Chem. (2019) 294:1643–51. 10.1074/jbc.TM118.004156 30710012 PMC6364759

[32] JJ N. Enzyme catalysis: The serine protases. Nat Educ. (2010) 3:21. 10.1002/iub.186

[33] Patel S Homaei A El-Seedi HR Akhtar N. Cathepsins: proteases that are vital for survival but can also be fatal. Biomed Pharmacother. (2018) 105:526–32. 10.1016/j.biopha.2018.05.148 29885636 PMC7172164

[34] Platt MO Ankeny RF Jo H. Laminar shear stress inhibits cathepsin l activity in endothelial cells. Arteriosclerosis Thrombosis Vasc Biol. (2006) 26:1784–90. 10.1161/01.ATV.0000227470.72109.2b 16709945

[35] Platt MO Shockey WA. Endothelial cells and cathepsins: biochemical and biomechanical regulation. Biochimie. (2016) 122:314–23. 10.1016/j.biochi.2015.10.010 26458976 PMC4747805

[36] Munro MJ Wickremesekera AC Davis PF Marsh R Tan ST Itinteang T. Renin-angiotensin system and cancer: A review. Integr Cancer Sci Therap. (2017) 10:745–59. 10.15761/ICST.1000231

[37] Lutgens SPM Cleutjens KBJM Daemen MJAP Heeneman S. Cathepsin cysteine proteases in cardiovascular disease. FASEB J. (2007) 21:3029–41. 10.1096/fj.06-7924com 17522380

[38] Pišlar A Kos J. Cysteine cathepsins in neurological disorders. Mol Neurobiol. (2014) 49:1017–30. 10.1007/s12035-013-8576-6 24234234

[39] Glowinski S Worowski K. Cathepsin D activity and protein degradation products content in the walls of varicose veins of the lower limbs. Eur Surg Res. (1981) 13:243–6. 10.1159/000128190 7262138

[40] Itinteang T Chudakova DA Dunne JC Davis PF Tan ST. Expression of cathepsins B, D, and G in infantile hemangioma. Front Surg. (2015) 2:26. 10.3389/fsurg.2015.00026 26137466 PMC4470331

[41] Tan DK Brasch RH Van Schaijik WB Armstrong FJ Marsh TR Davis TP. Expression and localization of cathepsins B, D, and G in Dupuytren's disease. Plast Reconstr Surg - Global Open. (2018) 6:e1686. 10.1097/GOX.0000000000001686 29616179 PMC5865920

[42] Paterson C Lee VMY Brasch HD van Schaijik B Marsh R Tan ST. Expression of cathepsins B, D and G by the embryonic stem cell-like population within human keloid tissues and keloid-derived primary cell lines. Plast Reconstr Surg. (2019) 144:1. 10.1097/PRS.0000000000006275 31764649

[43] Kim Y-W Lee B-B. Epidemiologic aspect of congenital vascular malformation. In: Kim Y-W, Lee B-B, Yakes WF, Do Y-S, editors. Congenital Vascular Malformations: A Comprehensive Review of Current Management. Berlin: Springer (2017). p. 31–4. 10.1007/978-3-662-46709-1_5

[44] McMillan K Dunphy L Nishikawa H Monaghan A. Experiences in managing arteriovenous malformations of the head and neck. Bri J Oral Maxillofac Surg. (2016) 54:643–7. 10.1016/j.bjoms.2016.03.020 27066717

[45] Greene AK Goss JA. Vascular anomalies: From a clinicohistologic to a genetic framework. Plast Reconstr Surg. (2018) 141:709e−17e. 10.1097/PRS.0000000000004294 29697621 PMC5922803

[46] Burrows P. Angiogenesis and Vascular Malformations In: Congenital Vascular Malformations [Internet]. Berlin: Springer (2017).

[47] Liu C-L Guo J Zhang X Sukhova GK Libby P Shi G-P. Cysteine protease cathepsins in cardiovascular disease: from basic research to clinical trials. Nat Rev Cardiol. (2018) 15:351–70. 10.1038/s41569-018-0002-3 29679024

[48] Siklos M BenAissa M Thatcher GRJ. Cysteine proteases as therapeutic targets: does selectivity matter? A systematic review of calpain and cathepsin inhibitors. Acta Pharm Sin B. (2015) 5:506–19. 10.1016/j.apsb.2015.08.001 26713267 PMC4675809

[49] Hook G Jacobsen JS Grabstein K Kindy M Hook V. Cathepsin B is a new drug target for traumatic brain injury therapeutics: Evidence for E64d as a promising lead drug candidate. Front Neurol. (2015) 6:178. 10.3389/fneur.2015.00178 26388830 PMC4557097

[50] Aoki T Kataoka H Ishibashi R Nozaki K Hashimoto N. Cathepsin B, K, and S are expressed in cerebral aneurysms and promote the progression of cerebral aneurysms. Stroke. (2008) 39:2603–10. 10.1161/STROKEAHA.107.513648 18635848

[51] Li W Peng H Seth DM Feng Y. The prorenin and (pro)renin receptor: New players in the brain renin-angiotensin system? Int J Hypertens. (2012) 2012:290635. 10.1155/2012/290635 23316344 PMC3536329

[52] Nguyen Dinh Cat A Touyz RM. A new look at the renin-angiotensin system-focusing on the vascular system. Peptides. (2011) 32:2141–50. 10.1016/j.peptides.2011.09.010 21945916

[53] Levy BI. How to explain the differences between renin angiotensin system modulators. Am J Hypertens. (2005) 18:134s−41. 10.1016/j.amjhyper.2005.05.005 16125050

[54] Tan CE Itinteang T Leadbitter P Marsh R Tan ST. Low-dose propranolol regimen for infantile haemangioma. J Paediatr Child Health. (2015) 51:419–24. 10.1111/jpc.12720 25187156

[55] Tan ST Itinteang T Day DJ O'Donnell C Mathy JA Leadbitter P. Treatment of infantile haemangioma with captopril. Br J Dermatol. (2012) 167:619–24. 10.1111/j.1365-2133.2012.11016.x 22533490

[56] Urbich C Heeschen C Aicher A Sasaki K-i Bruhl T Farhadi MR. Cathepsin L is required for endothelial progenitor cell-induced neovascularization. Nature Med. (2005) 11:206–13. 10.1038/nm1182 15665831

[57] Zhou Z Wang Y Bryant SH. Computational analysis of the cathepsin B inhibitors activities through LR-MMPBSA binding affinity calculation based on docked complex. J Comput Chem. (2009) 30:2165–75. 10.1002/jcc.21214 19242965 PMC2735608

[58] Li Y-Y Fang J Ao G-Z. Cathepsin B and L inhibitors: a patent review (2010 - present). Expert Opin Therap Patents. (2017) 27:643–56. 10.1080/13543776.2017.1272572 27998201

[59] Dumas J Brittelli D Chen J Dixon B Hatoum-Mokdad H Konig G. Synthesis and structure activity relationships of novel small molecule cathepsin D inhibitors. Bioorg Med Chem Lett. (1999) 9:2531–6. 10.1016/S0960-894X(99)00433-3 10498202

[60] Huo S Wang J Cieplak P Kollman PA Kuntz ID. Molecular dynamics and free energy analyses of cathepsin D-inhibitor interactions: insight into structure-based ligand design. J Med Chem. (2002) 45:1412–9. 10.1021/jm010338j 11906282

[61] Greco MN Hawkins MJ Powell ET Almond HR Jr Corcoran TW. Nonpeptide inhibitors of cathepsin G: optimization of a novel beta-ketophosphonic acid lead by structure-based drug design. J Am Chem Soc. (2002) 124:3810–1. 10.1021/ja017506h 11942800

[62] Swedberg JE Li CY de Veer SJ Wang CK Craik DJ. Design of potent and selective cathepsin G inhibitors based on the sunflower trypsin inhibitor-1 scaffold. J Med Chem. (2017) 60:658–67. 10.1021/acs.jmedchem.6b01509 28045523

[63] Ravish I Raghav N. Curcumin as inhibitor of mammalian Cathepsin B, Cathepsin H, acid phosphatase and alkaline phosphatase: a correlation with pharmacological activities. Med Chem Res. (2014) 23:2847–55. 10.1007/s00044-013-0872-1

[64] Fox C Cocchiaro P Oakley F Howarth R Callaghan K Leslie J. Inhibition of lysosomal protease cathepsin D reduces renal fibrosis in murine chronic kidney disease. Sci. Rep. (2016) 6:20101. 10.1038/srep20101 26831567 PMC4735715

[65] Rawlings N Barrett A. Introduction: aspartic and glutamic peptidases and their clans. Handbook of Proteolytic Enzymes. (2013) 1:3–19. 10.1016/B978-0-12-382219-2.00001-6

[66] Stein RL Strimpler AM. Slow-binding inhibition of chymotrypsin and cathepsin G by the peptide aldehyde chymostatin. Biochemistry. (1987) 26:2611–5. 10.1021/bi00383a030 3607037

[67] Allegri P Mastromarino A Neri P. Management of chronic anterior uveitis relapses: efficacy of oral phospholipidic curcumin treatment. Long-term follow-up. Clin Ophthalmol. (2010) 4:1201–6. 10.2147/OPTH.S13271 21060672 PMC2964958

[68] Maulina T Diana H Cahyanto A Amaliya A. The efficacy of curcumin in managing acute inflammation pain on the post-surgical removal of impacted third molars patients: A randomised controlled trial. J Oral Rehab. (2018) 45:677–83. 10.1111/joor.12679 29908031

[69] Hatcher H Planalp R Cho J Torti FM Torti SV. Curcumin: from ancient medicine to current clinical trials. Cell Mol Life Sci. (2008) 65:1631–52. 10.1007/s00018-008-7452-4 18324353 PMC4686230

[70] Kuttan R Sudheeran PC Josph CD. Turmeric and curcumin as topical agents in cancer therapy. Tumori. (1987) 73:29–31. 10.1177/030089168707300105 2435036

