# Supplementary material for: Expression of Cathepsins B, D, and G in Extracranial Arterio-Venous Malformation
Source: Front Surg. 2021 Aug 2;8:676871. doi: 10.3389/fsurg.2021.676871 (PMC8367294; doi:10.3389/fsurg.2021.676871)
Supplement: Supplementary file 1 [file Data_Sheet_1.docx]

Supplementary Material

**Supplementary Table 1. Patient demographics and anatomic location of their arterio-venous malformation**

| **Patient** | **Age*** | **Sex** | **Anatomical Location** |
| --- | --- | --- | --- |
| 1 | 22 | F | Forehead/scalp |
| 2 | 32 | F | Lower lip/chin |
| 3 | 65 | F | Upper lip |
| 4 | 54 | F | Nose |
| 5 | 19 | M | Foot |
| 6 | 44 | F | Scalp/ear |
| 7 | 17 | F | Foot |
| 8 | 48 | M | Hand |
| 9 | 17 | M | Foot |
| 10 | 20 | M | Forearm |
| 11 | 17 | M | Foot |
| 12 | 58 | M | Hand |
| 13 | 53 | F | Hand |

*years; M, male; F, female

**Supplementary Table 2. Localization of cathepsins B, D and G in 13 arterio-venous malformation tissue samples**

| **Tissue sample** | **Cathepsin B** | **Cathepsin D** | **Cathepsin G** |
| --- | --- | --- | --- |
| 1 | Endothelium, media, stroma | Endothelium, stroma | Stroma |
| 2 | Endothelium, media | Endothelium, stroma | Stroma |
| 3 | Endothelium, media, stroma | Endothelium, stroma | Stroma |
| 4 | Endothelium, media, stroma | Endothelium, stroma | Stroma |
| 5 | Endothelium, media, stroma | Endothelium, stroma | Stroma |
| 6 | Endothelium, media, stroma | Endothelium, stroma | Stroma |
| 7 | Endothelium, media, stroma | Endothelium, stroma | Stroma |
| 8 | Endothelium, media | Endothelium, stroma | Stroma |
| 9 | Endothelium, media | Stroma | Stroma |
| 10 | Endothelium, media, stroma | Endothelium, stroma | Stroma |
| 11 | Endothelium, p media, stroma | Stroma | Stroma |
| 12 | Endothelium, media, stroma | Endothelium, stroma | Stroma |
| 13 | Endothelium, media | Endothelium, stroma | Stroma |


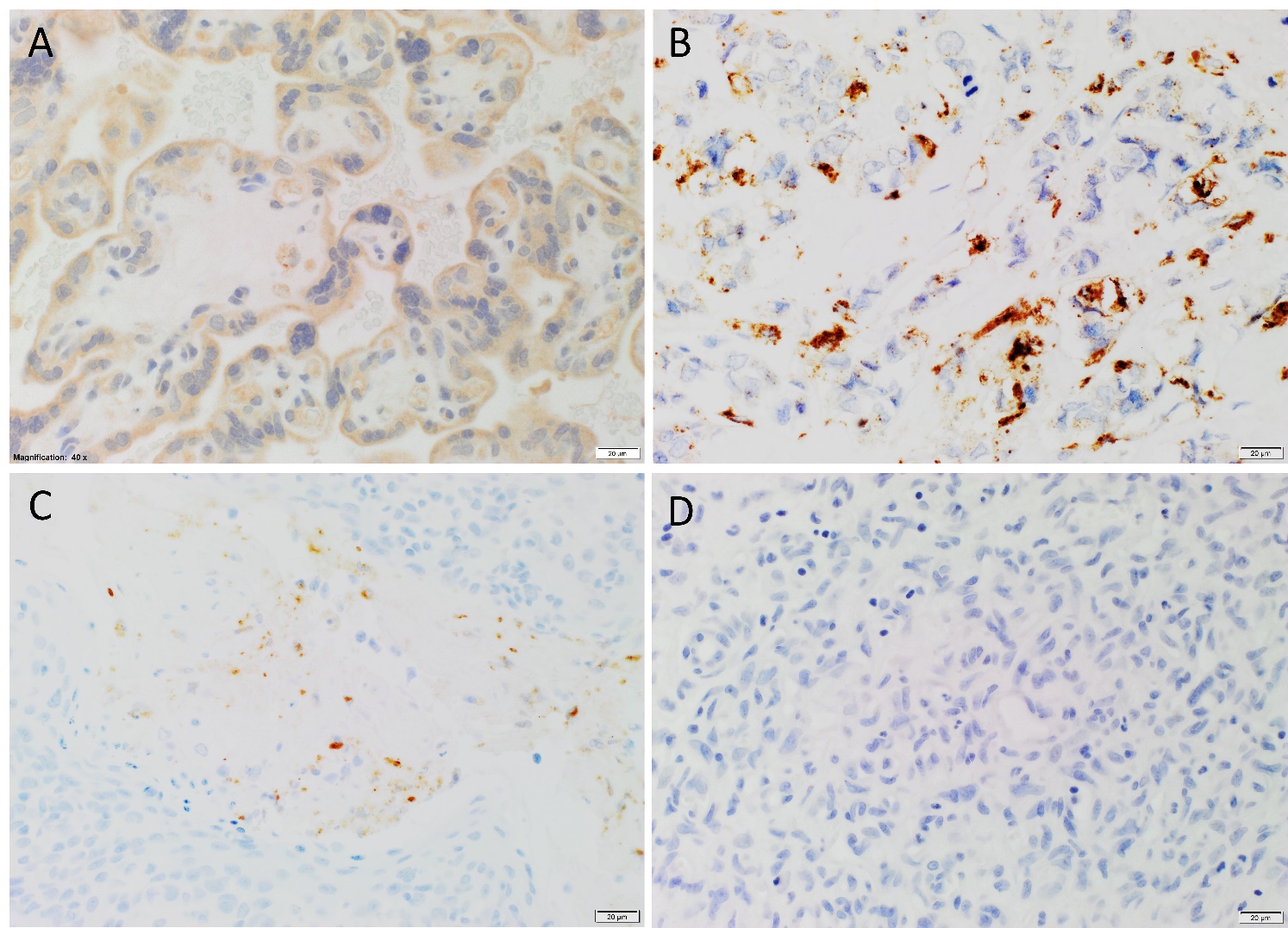


**Supplementary Figure 1** Representative immunohistochemical-stained sections of human control tissues showing expected staining pattern for cathepsin B (**A**, brown) on placenta, cathepsin D (**B**, brown) on breast carcinoma, and cathepsin G (**C**, brown) on tonsil. Negative controls performed on a section of arterio-venous malformation matched to immunohistochemical-stained sections of isotype controls for mouse and rabbit showed no staining, confirming the specificity of the secondary antibody (**D**). Nuclei were counterstained with hematoxylin (**A**-**D**, blue). Original magnification: 400x


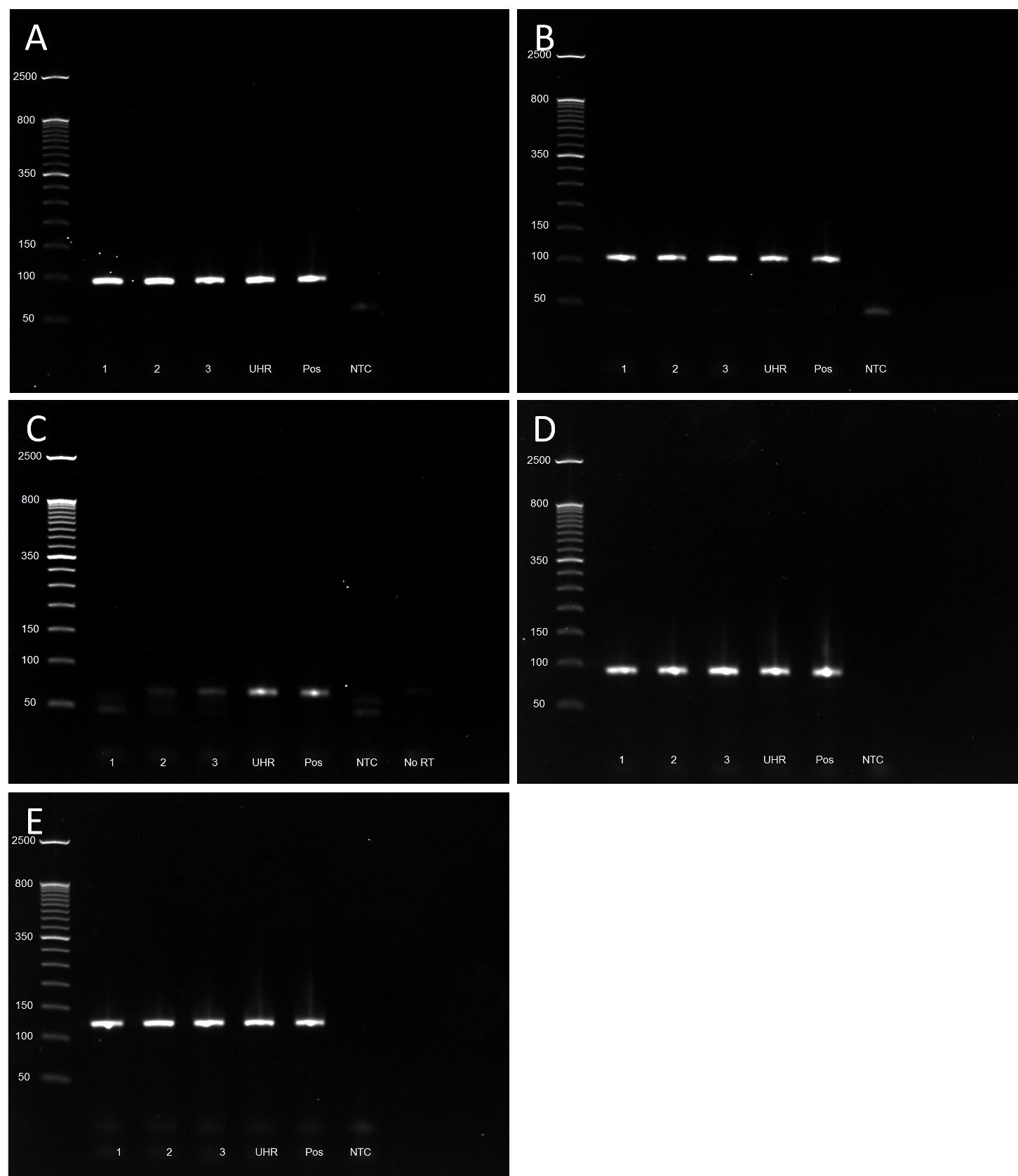
**Supplementary Figure 2** RT-qPCR amplification products from three arterio-venous malformation (AVM)-derived primary cells were checked using agarose gel electrophoresis to confirm probe specificity. Cathepsin B (**A**, 94bp), cathepsin D (**B**, 103bp) and cathepsin G (**C**, 62bp) and reference genes PUM1 (**D**, 89bp) and GAPDH (**E**, 122bp) were checked. Lanes 1-3 refer to respective AVM-derived primary cell lines; UHR, universal human reference; Pos, positive control (tonsil); NTC, no template control (RNase-free water) to confirm no contamination; No RT, reverse transcriptase negative control for primers that may detect genomic DNA.


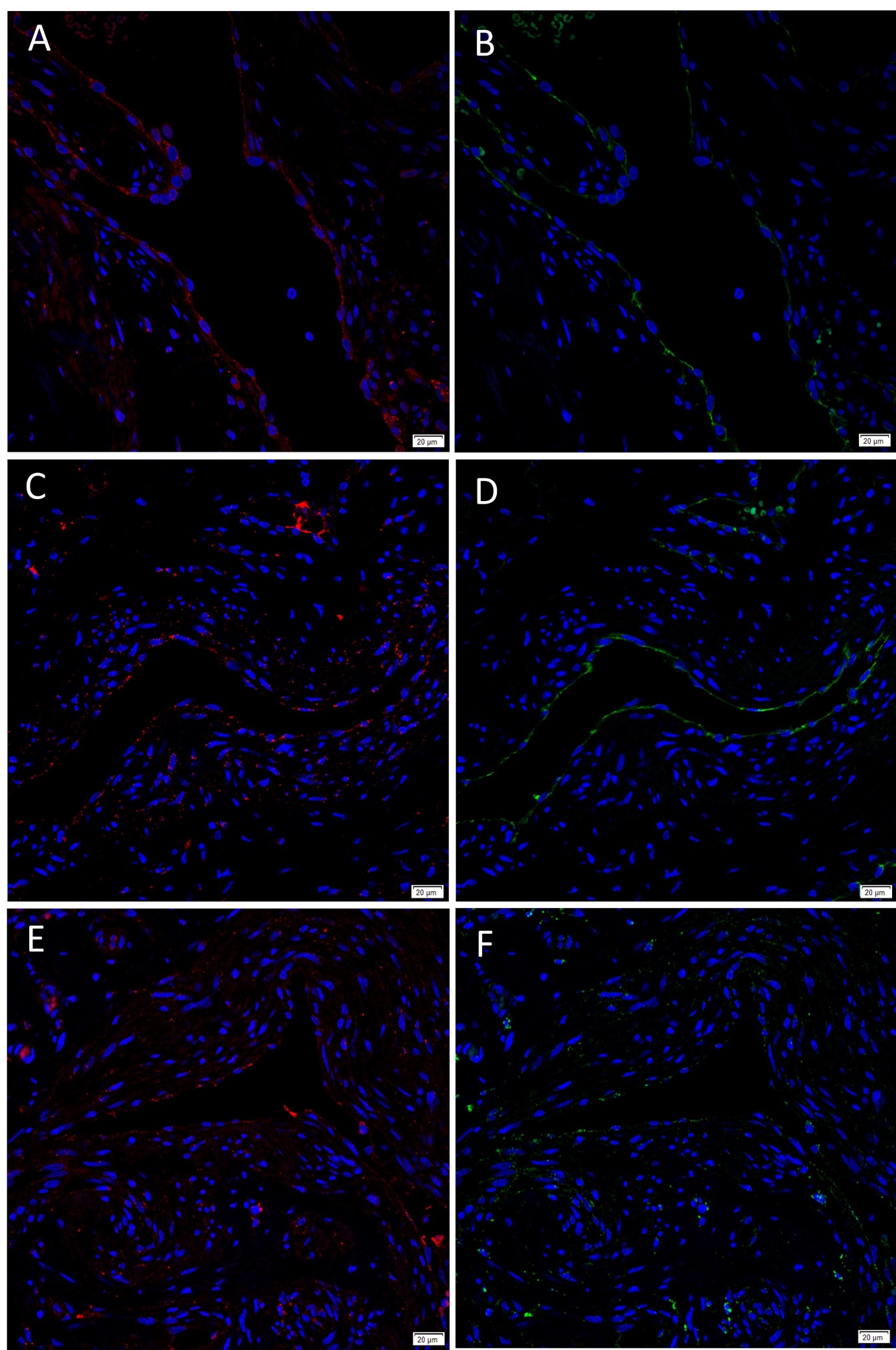


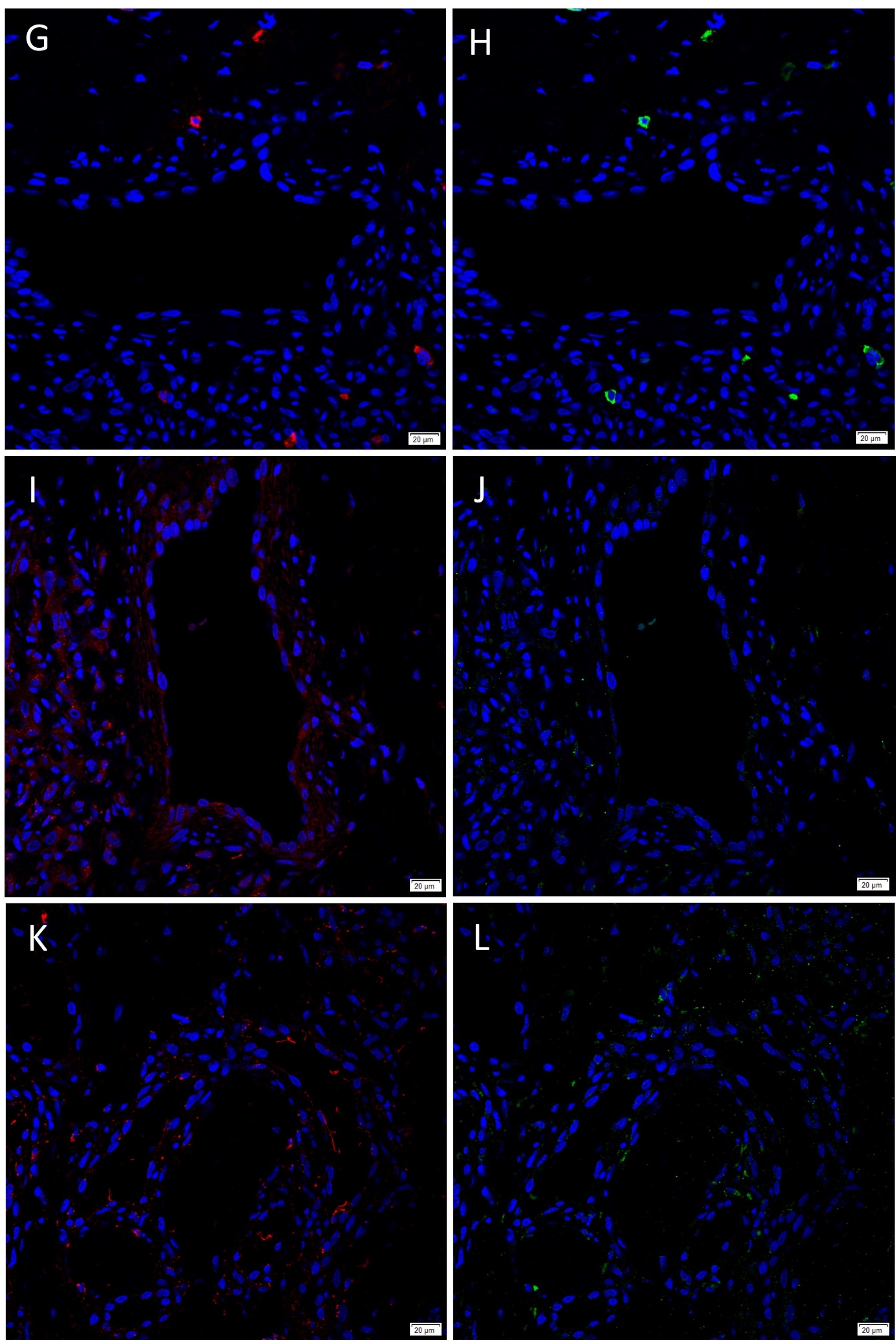


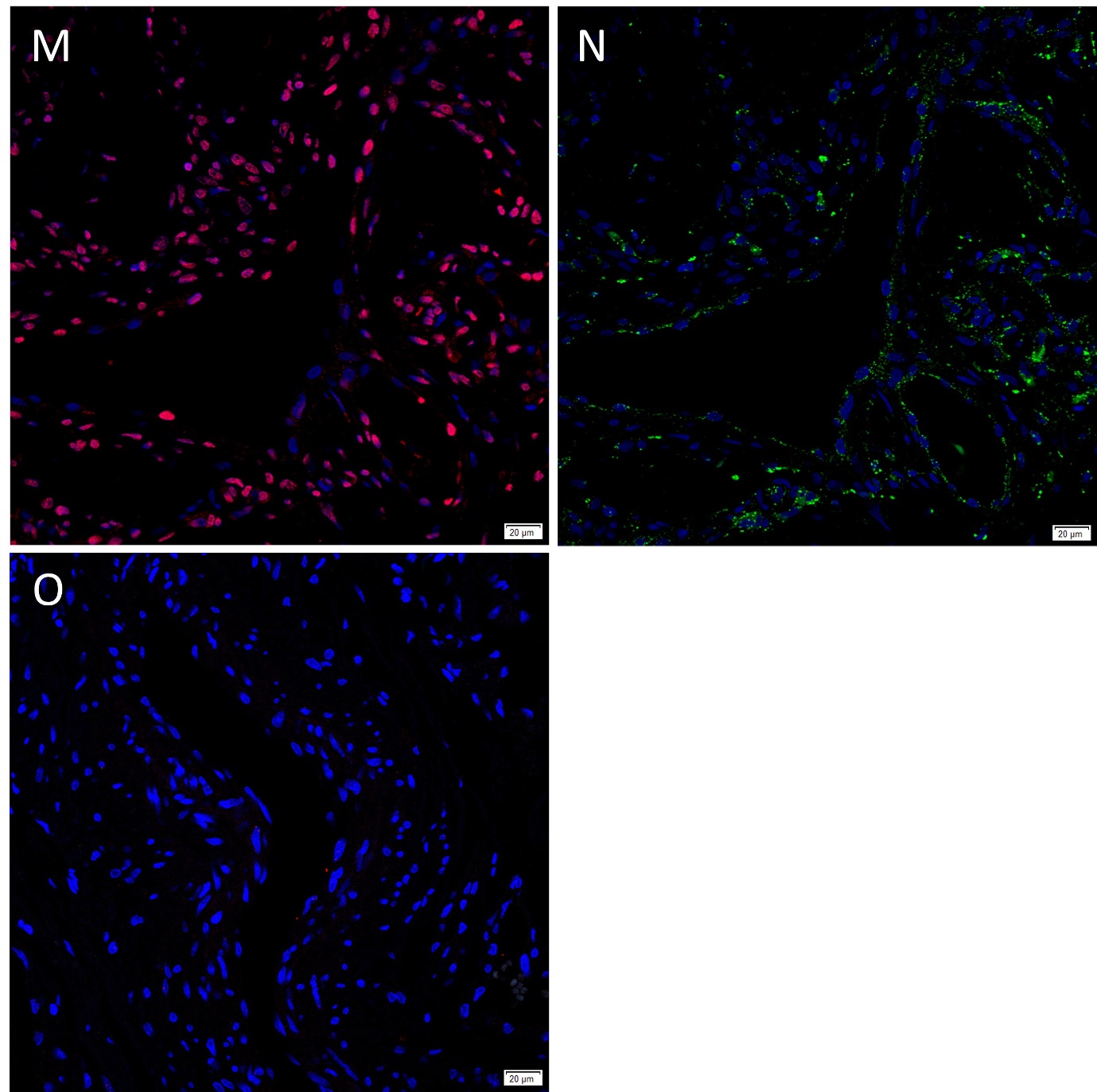


**Supplementary Figure 3** Split images of immunofluorescence-stained sections of arterio-venous malformation (AVM) tissue samples demonstrated in Figure 4, showing the expression of cathepsin B (**A**, red) and CD31 (**B**, green); cathepsin D (**C**, red) and CD31 (**D**, green); cathepsin D (**E**, red) and cathepsin B (**F**, green); cathepsin G (**G**, red) and chymase (**H**, green); cathepsin B (**I,** red) and OCT4 (**J**, green); cathepsin D (**K**, red) and OCT4 (**L**, green); and SOX2 (**M**, red) and cathepsin B (**N**, green). Negative control (**O**) performed on a section of AVM showed the specificity of the fluorescent secondary antibodies. Cell nuclei were counterstained with 4′,6-diamidino-2-phenylindole (**A**-**O**, blue). Original magnification: 400x.


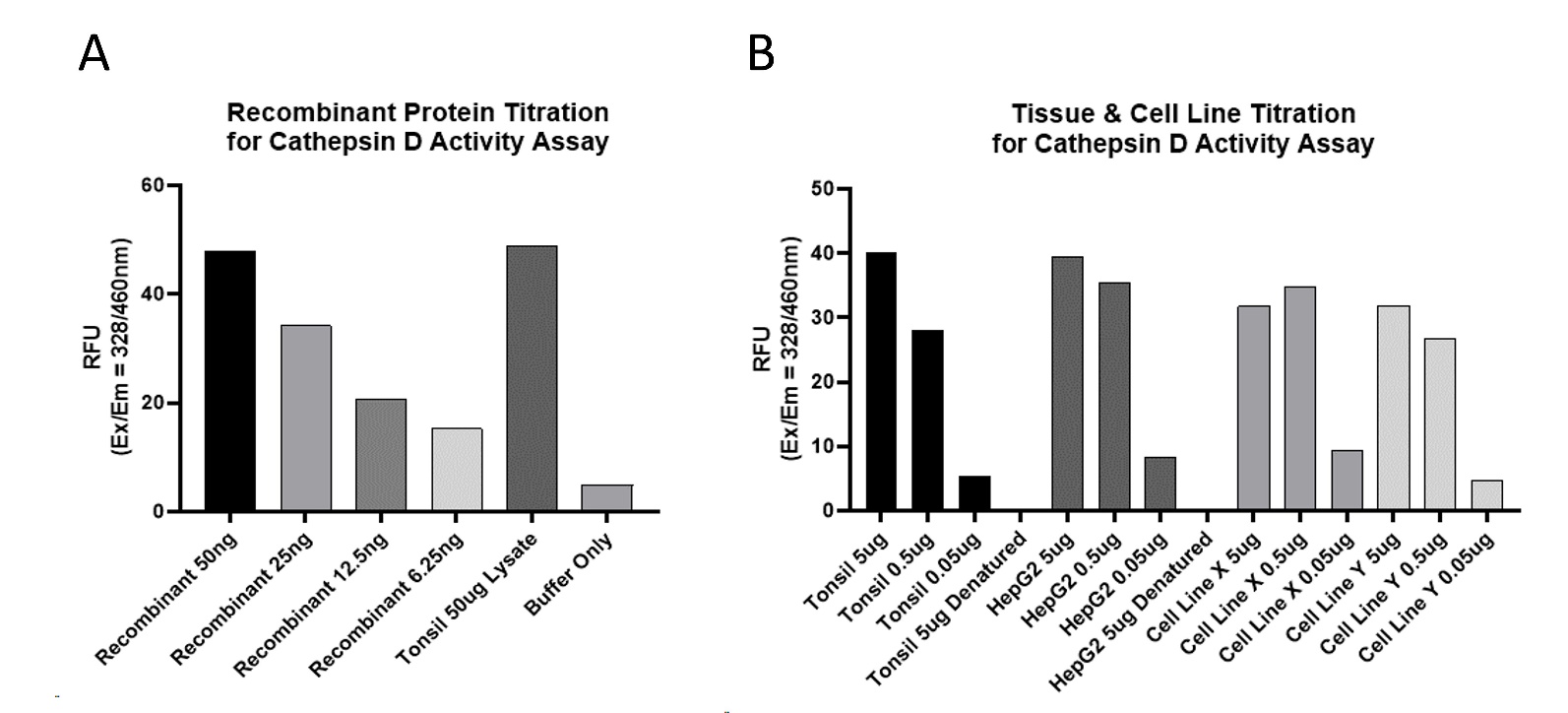


**Supplementary Figure 4** Cathepsin D activity assays. Due to a very small dynamic range for the cathepsin D activity assay, a titration was carried out using a recombinant cathepsin D protein to validate the assay (**A**). A further titration assay was carried out using tissue and cell line samples to establish the appropriate amount of protein to add for a valid assay result (**B**).
